# Datasets for distributed denial-of-service detection in healthcare internet of things environments

**DOI:** 10.1016/j.dib.2025.112222

**Published:** 2025-11-01

**Authors:** Mirza Akhi, Ciarán Eising, Lubna Luxmi Dhirani

**Affiliations:** Department of Electronic and Computer Engineering, University of Limerick, Limerick, V94 T9PX, Ireland

**Keywords:** Cybersecurity, Cooja simulator, Ns-3 simulator, IoT traffic features, Network simulation, Traffic analysis, Anomaly detection, Machine Learning for cyber defense

## Abstract

The growing number of Internet of Things (IoT) devices in healthcare settings raises critical concerns about security, particularly in defending against Distributed Denial-of-Service (DDoS) attacks. These attacks can cause operational downtime in IoT environments. To mitigate DDoS-based attacks, advanced defense-in-depth strategies and well-labeled datasets are required for Healthcare-IoT (H-IoT), IoT, and other distributed computing contexts. This article presents two labeled datasets, ***UL-ECE-MQTT-DDoS-H-IoT2025*** and ***UL-ECE-UDP-DDoS-H-IoT2025***, generated by simulating realistic traffic patterns under both normal and DDoS conditions using the Cooja and ns-3 simulators. In Cooja, the raw dataset records healthcare-specific Message Queuing Telemetry Transport (MQTT) traffic (e.g., simulated oxygen level of 100 %) randomly generated by emulated H-IoT sensors. It also includes message counts and network metadata that enable detailed analysis across both application and network layers. In ns-3, the raw data comprises 5G-enabled H-IoT network traces from all nodes, capturing timestamps, payload size, and the header details of the User Datagram Protocol (UDP). Existing benchmark datasets mainly consist of generic network traffic attributes, including packet IDs, protocol types, and timestamps. In contrast, the proposed datasets address this gap by incorporating H-IoT-specific communication parameters that closely resemble real-world conditions, such as node-level message counts and monitoring frequencies. This inclusion provides a realistic representation of communication patterns for security and performance research in H-IoT. The datasets enable detailed analysis of key features for detecting DDoS threats, including UDP flood variants extending beyond the H-IoT domain. This characteristic makes them directly usable for developing, testing, and comparing machine learning (ML) and deep learning (DL) models across diverse IoT security contexts. The MQTT-based dataset is derived from a 5-hour simulation run using the Cooja simulator, which emulates wearable sensors such as body temperature, heart rate, and oxygen saturation. In this setup, normal H-IoT nodes transmit data to the server at 60-second intervals, while DDoS-affected nodes publish data at 20-second intervals to simulate a higher transmission frequency. The UDP-based dataset is derived from a 120-second simulation conducted using the ns-3 simulator, which simulates a 5G-enabled H-IoT environment. In this scenario, normal and malicious nodes transmit data at 124 kbps and 248 kbps, respectively. Both datasets are processed from raw simulation logs converted into structured CSV files using Python scripts. The CSV files contain features such as timestamp, payload size, message frequency, and node-level communication statistics. The ***UL-ECE-MQTT-DDoS-H-IoT2025*** and ***UL-ECE-UDP-DDoS-H-IoT2025*** datasets contain approximately 20,080 and 99,887 records, respectively. The primary objective of creating these datasets is to enhance security in healthcare IoT ecosystems by enabling robust detection of advanced cyber threats. In line with this objective, the datasets support the development of ML/DL-based cybersecurity mechanisms. In addition, this resource forms a foundation for future research, motivating the creation of new datasets for emerging attack scenarios.

Specifications Table

**Table:** 

Subject	Computer Sciences
Specific subject area	Healthcare-Internet of Things (H-IoT) focuses on cybersecurity, distributed denial-of-service (DDoS) detection using machine learning (ML) methods
Type of data	Structured tabular data (Processed and Analyzed)
Data collection	Data is collected using two simulators, Cooja for MQTT and ns-3 for UDP. The MQTT-based dataset includes 14 normal and 5 DDoS-affected H-IoT nodes emulating wearable sensors (e.g., heart rate, body temperature) over Contiki OS 3.0. The UDP-based dataset simulates a 5G H-IoT environment with 14 clients, 5 DDoS-affected nodes, and 3 additional DDoS attackers introduced at 20 s with 10 s intervals. Simulations run in Oracle VirtualBox using Windows 11 and Ubuntu 24.10 on a system with Intel Core i5–12500H CPU, 16 GB RAM, and NVIDIA GeForce RTX GPU.
Data source location	University of Limerick, Limerick, Ireland Latitude: 52.6736° N, Longitude: 8.5721° W
Data accessibility	**Repository name:** Zenodo **Data identification number (DOI):** 10.5281/zenodo.15305814 **Direct URL to data:**https://zenodo.org/record/15305814
Related research article	M. Akhi, C. Eising, L.L. Dhirani, TCN-based DDoS detection and mitigation in 5G healthcare-IoT: A frequency monitoring and dynamic threshold approach, IEEE Access. 13 (2025) 12,709–12,733. https://doi.org/10.1109/ACCESS.2025.3531659

## 1 Value of the Data

• Privacy regulations and ethical concerns limit the ability to generate malicious data from physical Healthcare-IoT (H-IoT) devices (e.g., body temperature and oxygen saturation) [1,2]. As an alternative, synthetic data generated through simulation provides a practical way to continue cybersecurity research in H-IoT environments. It enables the creation of realistic DDoS attack scenarios where parameters like payload size, data traffic interval, and protocol type are systematically adjusted, capturing both regular and malicious traffic without compromising data security or violating privacy regulations (e.g., GDPR).
• Synthetic data provides a cost-effective solution that reduces reliance on both expensive physical H-IoT device-based records and the need for access to sensitive healthcare information. However, its simulated nature might not accurately capture the unpredictability of real-world settings, including device-to-device interactions, malfunctions, traffic across multiple protocols and dynamic sensor behaviors (e.g., fluctuating readings). This limitation should be considered when interpreting results for practical deployment. Nonetheless, the datasets remain a scalable, openly shareable, and manageable resource applicable across cyberattack scenarios in H-IoT contexts.
• Researchers, cybersecurity analysts, and data scientists working on anomaly detection and DDoS mitigation in H-IoT networks may utilize these datasets to develop, test, and validate ML and deep learning (DL) models across both MQTT and UDP protocols in H-IoT and broader IoT ecosystems. Additionally, educators and students may leverage these datasets for academic projects (e.g., Master’s theses) to analyze network security and IoT attacks using AI/ML techniques.
• The ***UL-ECE-MQTT-DDoS-H-IoT2025*** and ***UL-ECE-UDP-DDoS-H-IoT2025*** datasets enable assessment of detection accuracy by comparing the proposed models with existing anomaly detection techniques. The datasets also provide a basis for evaluating model generalization by comparing detection performance against other publicly available DDoS datasets.
• Analysis of variations in traffic parameters (e.g., packet interval, payload size, attack duration) and their impact on key features, such as total messages, node-specific message counts, and transmission frequency in H-IoT networks, can provide deeper insights into network behavior. The datasets also provide ample and valuable data for evaluating intrusion detection systems in network security, contributing to ongoing efforts to address emerging cybersecurity threats. Prior research has demonstrated the importance of such features in improving ML/DL-based detection performance [3,4].
• In the broader IoT environment, these open-access DDoS attack datasets serve as a valuable resource, filling a critical gap by enabling protocol-specific analysis with controlled configurations (e.g., traffic interval).

## 2 Background

Creating realistic attack scenarios on H-IoT devices is challenging in real-world settings due to privacy, ethical, and safety concerns, as well as limited access to physical infrastructure for testing vulnerabilities. However, continued research in this domain requires analyzing how IoT nodes interact under normal and attack conditions. In such cases, simulated or synthetic health-related data are essential for analyzing device-to-device interactions and protocol-specific vulnerabilities. Moreover, synthetic data generation facilitates the development of ML/DL models in domains where access to real-world data is limited or restricted. To address this gap, we generate two datasets over the MQTT and UDP protocols using the Cooja and ns-3 simulators in H-IoT environments [5,6]. The *UL-ECE-MQTT-DDoS-H-IoT2025* and *UL-ECE-UDP-DDoS-H-IoT2025* datasets support our original research article, which presents a detection and mitigation approach using a deep learning model, the Temporal Convolutional Network (TCN) [7]. The original research article mainly focuses on implementing and evaluating the proposed method. However, this data article provides detailed documentation of the simulation setup, including raw and processed datasets, as well as the Python scripts used for data preprocessing. Publishing the datasets as an article with a DOI affirms authenticity, ensures wider accessibility, and strengthens the contribution of the original research.

## 3 Data Description

This section describes the ***UL-ECE-MQTT-DDoS-H-IoT2025*** and ***UL-ECE-UDP-DDoS-H-IoT2025*** datasets of the UL-ECE-DDoS-H-IoT-Datasets2025 [8]. It details the folder structure, data files, and corresponding preprocessing scripts located under the MQTT and UDP directories. Each dataset consists of three components: raw simulation logs, processed CSV files containing labeled traffic records, and Python scripts for preprocessing these records. It describes the key features of the ***UL-ECE-MQTT-DDoS-H-IoT2025*** dataset and presents a sample table to illustrate its processed data format. It highlights the 13 key features of the ***UL-ECE-UDP-DDoS-H-IoT2025*** dataset and presents a sample table for these parameters. It also explains the 4 features excluded from the TCN model’s training phase. Finally, it discusses a comparison with existing datasets (e.g., CIC-IDS2017).

### 3.1 Dataset details for MQTT

This section details the MQTT folder of the **UL-ECE-DDoS-H-IoT-Datasets2025** [8], including the processed CSV file (UL-ECE-MQTT-DDoS-H-IoT2025), raw communication logs, and the Python script used for preprocessing.

The file **UL-ECE-MQTT-DDoS-H-IoT2025.csv** contains labeled MQTT traffic data generated using the Cooja simulator in a healthcare-IoT context. This CSV file includes both normal and DDoS attack-affected traffic records, where each row represents data from a single H-IoT node and contains 4 feature columns. The corresponding raw traffic is stored in **UL-ECE-MQTT-DDoS-H-IoT2025-raw.txt**, located in the same MQTT folder, which contains the original MQTT communication logs captured during the simulation.

Additionally, the MQTT folder contains the file data_preprocessing_ddos_mqtt_cooja.py, which is the Python script used to preprocess the raw MQTT traffic logs. It generates a labeled CSV file containing extracted features for ML operations. All files described above are archived on Zenodo [8].

### 3.2 Data preprocessing and feature extraction for MQTT

We generate raw MQTT communication logs in the Cooja simulator and preprocess the obtained raw data using the Python script (data_preprocessing_ddos_mqtt_cooja.py) to produce the labeled dataset. This preprocessing pipeline parses the simulation output, cleans the data, and extracts key features. The resulting dataset comprises 4 features:

**Node IDs:** Numeric identifiers assigned by the Cooja simulator to each simulated sensor node, used to distinguish individual H-IoT devices or sensors in the network.

**Total Messages:** This feature tracks the cumulative number of messages recorded at each observation during data transmission in the H-IoT network. The total message count T is computed using the following formula:

(1) $$T=\sum_{i=1}^{N}M_{t}$$

where $M_{i}$represents a message indicator at the $i^{th}$ observation. Further details of this metric and its derivation are available in our related journal publication [7].

**Total Messages Each Node:** This feature represents the total number of messages transmitted by each individual node in the H-IoT network. It reflects the communication activity of a node by summing up all messages it transmits throughout the data transmission period. The total messages sent by node i are calculated using the following expression:

(2) $$total\_messages\_each\_node_{i}=\sum_{j=1}^{N}M_{nodei,j}$$

where $M_{nodei,j}$​ denotes whether node i transmitted a message at the $j^{\mathrm{th}}$ logged transmission.

A detailed explanation of the feature derivation and thresholding logic can be found in our related journal publication [7].

**Message Frequency Proportion (frequency):** This feature represents the proportion of messages sent by each node as a percentage of the total number of messages in the network. It reflects the relative activity level of each node and is computed using the following equation:

(3) $$frequency\;\left(\%\right)=\left(\frac{M_{node_{i}}}{M_{total}}\right)\times 100$$

where $M_{node_{i}}$ is the total number of messages transmitted by node i and $M_{total}$ is the cumulative number of messages across all nodes. This feature plays a crucial role in identifying DDoS-affected nodes, as an abnormally high frequency value may indicate anomalous or malicious behavior. A threshold-based mechanism is applied to flag nodes that exceed a frequency of 5 %, as illustrated in the sample dataset provided in Table 1. Detailed discussion of the thresholding method and feature behavior is available in our related journal publication [7].

**Table 1:** Example entries from the ***UL-ECE-MQTT-DDoS-H-IoT2025*** dataset highlighting key traffic features.

ids	total_message	total_message_each_node	frequency
11	2476	90	3.634894991922456
18	2477	252	10.173597093257973
8	2478	89	3.5916061339790155
18	2479	253	10.205728116175877
16	2480	247	9.959677419354838
20	2481	269	10.842402257154374
14	2482	93	3.7469782433521353
15	2483	68	2.738622633910592
10	2484	144	5.797101449275362
8	2485	90	3.6217303822937628

***Note:*** The **ids** mentioned in the first column of the table correspond to the **node** column in the dataset, which represents unique identifiers for each node in the network.

### 3.3 Dataset details for UDP

This section outlines the contents of the **UDP** directory within the **UL-ECE-DDoS-H-IoT-Datasets2025** [8], which includes the processed CSV file, and the Python script used during data preprocessing.

The file **UL-ECE-UDP-DDoS-H-IoT2025.csv** contains UDP-based network traffic data labeled as either normal or DDoS attack-affected in a healthcare-IoT context. The raw simulation log file corresponding to this dataset is generated using the ns-3 simulator. It is not included in the repository due to file-size limitations; it remains available upon request.

The directory also includes the Python script (data_preprocessing_ddos_udp_ns_3.py), which transforms the raw simulation output into the structured CSV format.

The **README.md** file in the root directory provides an overview of the MQTT and UDP datasets, detailing their structure, including files, and usage instructions.

### 3.4 Data preprocessing and feature extraction for UDP

Raw UDP communication logs from the ns-3 simulator are processed to build the labeled dataset. The preprocessing pipeline (data_preprocessing_ddos_udp_ns_3.py) applies a rule-based parser and a 5-second sliding-window aggregation method. It performs data cleaning and extracts features specific to UDP traffic. Within this pipeline, timestamps are extracted to calculate elapsed times, and per-node message counts are continuously tracked. Categorical fields (protocol, source IP, destination IP) are encoded as numeric codes, and the corresponding original string values (e.g., source_ip_des = 7.0.0.13) are also retained in separate columns for reference. The pipeline computes both running message frequencies and the running mean and aggregates monitoring totals from the last 5 s to determine the monitoring frequency. If the 5-second monitoring frequency is greater than or equal to the running mean frequency, the labeling rule assigns a binary outcome label, malicious = 1 and normal = 0. The resulting CSV file **UL-ECE-UDP-DDoS-H-IoT2025.csv** contains key time-based parameters (e.g., timestamp, time elapsed) alongside node-specific parameters such as node ID, protocol type, IP addresses, payload size, and message frequency.

The ***UL-ECE-UDP-DDoS-H-IoT2025*** dataset contains 17 features; however, our published journal paper [7] utilizes 13 features to achieve optimal accuracy with the TCN model. The published paper [7] also discusses these 13 features with detailed equations. In this article, the features are presented as in the ***UL-ECE-UDP-DDoS-H-IoT2025*** dataset shown in Table 2.

**Table 2:** Representative sample entries from the ***UL-ECE-UDP-DDoS-H-IoT2025*** dataset.

ids	time	protocol	source_ip	destination_ip	payload_size	total_messages	total_messages_same_node	frequency	mean_frequency	monitoring_total_ messages	monitoring_total_messages_same_node	monitoring_frequency
15	108.6924	0	3	0	512	89,981	3291	3.6574	5.4325	4400	152	3.4545
16	108.6924	0	4	0	512	89,982	3291	3.6573	5.4325	4400	152	3.4545
17	108.6924	0	5	0	512	89,983	3291	3.6573	5.4325	4400	152	3.4545
18	108.6924	0	6	0	512	89,984	3291	3.6573	5.4325	4400	152	3.4545
19	108.6924	0	7	0	512	89,985	6582	7.3145	5.4325	4400	303	6.8863
20	108.6924	0	8	0	512	89,986	6582	7.3144	5.4325	4400	303	6.8863
21	108.6924	0	9	0	512	89,987	6582	7.3143	5.4325	4400	303	6.8863
22	108.6924	0	10	0	512	89,988	6582	7.3143	5.4326	4400	303	6.8863
23	108.6924	0	11	0	512	89,989	6582	7.3142	5.4326	4400	303	6.8863
25	108.6994	0	13	0	512	89,990	4766	5.2961	5.4326	4400	303	6.8863

***Note:*** The **ids** column corresponds to the **node_id** field in the **UL-ECE-UDP-DDoS-H-IoT2025.csv** dataset.

Moreover, while Table 2 presents the numerical values of the selected features, Table 3 lists these features in plain text along with their definitions. These concise definitions clarify the meaning and role of each feature in detecting DDoS attacks across H-IoT, general IoT, and intrusion scenarios.

**Table 3:** The 13 selected features from the ***UL-ECE-UDP-DDoS-H-IoT2025*** dataset are presented without underscores, alongside definitions relevant to DDoS detection.

Feature Name	Definition
Node IDs	Unique identifiers are assigned to each node in the network topology.
Time Elapsed	The time elapsed indicates the difference between the current timestamp and the initial timestamp.
Protocol	Numerical code representing the communication protocol (0 for UDP in this dataset).
Source IP Address	Unique IP address of each node that transmits packets to the destination server.
Destination IP Address	Node IDs transmit data to the same destination node (e.g., server at 7.0.0.2, labeled as 0 for all H-IoT nodes in the dataset).
Payload Size	Size of each packet in bytes (e.g., 512).
Total Messages	Cumulative count of messages sent by the node.
Total Messages from Same Node	Total number of messages transmitted by a specific source node.
Frequency	Percentage of messages from the current node ID compared to the total messages processed until the current log line.
Mean Frequency	Average percentage of messages from all nodes up to the current log line.
Monitoring Total Messages	It is calculated by summing the messages monitored from each H-IoT node in the network within a 5-second interval during the simulation.
Monitoring Total Messages from Same Node	Rate of messages from a single H-IoT node, calculated using total messages sent, simulation time, and monitoring interval.
Monitoring Frequency	The proportion of messages from the same H-IoT node within the last 5 s compared with all messages in that period. This message-based proportion helps detect newly appearing nodes, which may initially have a lower message frequency than established ones.

The remaining 4 features (timestamp, protocol_des, source_ip_des, and destination_ip_des) store the original string values of timestamps, protocol types, and IP addresses to support querying and manual review of the generated dataset, as shown in Table 4. The ids column, one of the 13 selected features retained for model training, is also included in Table 4. It is presented primarily to give context for the excluded features and to illustrate their values for specific H-IoT nodes. These 4 features are retained for reference purposes in the ***UL-ECE-UDP-DDoS-H-IoT2025*** dataset; however, they are excluded from the TCN model’s training phase to improve generalization and maintain optimal accuracy [7]. The specific roles of these features and the rationale for their exclusion are described below.

**Table 4:** Representative sample entries of the 4 excluded features (timestamp, protocol_des, source_ip_des, and destination_ip_des) from the ***UL-ECE-UDP-DDoS-H-IoT2025*** dataset.

ids	timestamp	protocol_des	source_ip_des	destination_ip_des
15	108.709	ns3::Ipv4Header	7.0.0.13	7.0.0.2
16	108.709	ns3::Ipv4Header	7.0.0.14	7.0.0.2
17	108.709	ns3::Ipv4Header	7.0.0.15	7.0.0.2
18	108.709	ns3::Ipv4Header	7.0.0.16	7.0.0.2
19	108.709	ns3::Ipv4Header	7.0.0.17	7.0.0.2
20	108.709	ns3::Ipv4Header	7.0.0.18	7.0.0.2
21	108.709	ns3::Ipv4Header	7.0.0.19	7.0.0.2
22	108.709	ns3::Ipv4Header	7.0.0.20	7.0.0.2
23	108.709	ns3::Ipv4Header	7.0.0.21	7.0.0.2
25	108.716	ns3::Ipv4Header	7.0.0.23	7.0.0.2

***Note:*** The **ids** column is among the 13 selected features retained for model training and is not excluded.

**Timestamp:** This feature represents the simulation time when an H-IoT node sends a data packet to the server. While each record has a unique timestamp, the values primarily reflect the simulation period rather than meaningful attack-related patterns. As a result, the model may learn time-dependent correlations instead of genuine network traffic characteristics (e.g., packet size, message frequency). This introduces noise and bias without predictive value and degrades generalization. In our experiments, training the TCN model with this feature reduced accuracy; thus, it is not used in the final feature set.

**Protocol_des:** This feature contains the descriptive string ``ns3::Ipv4Header'' for the IPv4 header (e.g., the network layer packet structure, such as version (IPv4 vs. IPv6), source and destination IP address, etc.) in every record. Since this value is constant across all H-IoT node entries, it provides no variation for the model to learn from. Moreover, machine learning models cannot directly utilize raw string values without encoding. Any protocol differences (e.g., UDP vs. TCP, MQTT, or other protocols) are already captured by the numeric protocol feature (e.g., 0 for UDP). This allows detection if an external IoT node uses a different protocol. For this reason, protocol_des is omitted from the final set of features used for training.

**Source_ip_des:** This feature represents the dotted-decimal IP address of the source node (e.g., 7.0.0.13), which is already numerically encoded in the source_ip feature (e.g., 1, 2, 3). For example, taking the first row of Table 2, Table 4 for H-IoT node 15, if the log line shows /NodeList/15/ 7.0.0.13 > 7.0.0.2, the source IP is 7.0.0.13, which belongs to group 3 as shown in the tables. These groups are created in the same way for all H-IoT nodes. Hence, source_ip_des is excluded from the features used for model training to avoid redundancy.

**Destination_ip_des:** This feature contains the dotted-decimal IP address of the destination node, which is constant in this dataset (7.0.0.2, the server IP) for all records. Since it offers no variation for the model to learn from, and its numeric form is already represented in the destination_ip feature (e.g., 0 for all H-IoT nodes), destination_ip_des is not included in the features used for model training.

### 3.5 Comparison with existing datasets

DDoS attacks pose a significant threat within the cybersecurity landscape, prompting researchers to develop datasets that capture both normal and attack behaviors. Over the past ten years, several publicly available datasets have been used to evaluate DDoS detection techniques with advanced ML/DL models in IoT and H-IoT environments. These include broader network intrusion detection scenarios such as BoT-IoT, TON_IoT, and CICIoT2023 [9]. Table 5 provides a comparative overview of datasets, including CIC-IDS2017 [10], BoT-IoT [11], and WSN-DDoSAttack-H-IoT2023 [3], which are frequently used in ML/DL-based DDoS detection research. It also presents the detection accuracy achieved using the TCN model on the proposed UL-ECE-MQTT-DDoS-H-IoT2025 and UL-ECE-UDP-DDoS-H-IoT2025 datasets. The comparison highlights key aspects such as IoT/H-IoT focus, protocol, attack type, class type, model type, and accuracy.

**Table 5:** Comparison of existing public datasets with the proposed ***UL-ECE-MQTT-DDoS-H-IoT2025*** and ***UL-ECE-UDP-DDoS-H-IoT2025*** datasets, evaluated using the TCN model.

Ref.	Dataset	IoT/H-IoT Focus	Protocol	Attack Type	Class Type	Model	Accuracy (%)
[10] (2017)	CIC-IDS2017	✗	HTTP, HTTPS	DDoS	Binary	RF	99.31
[11] (2018)	BoT-IoT 2018	✓	Not specified	DoS/ DDoS	Binary	DT	100
[3] (2024)	WSN_DDoS_Attack_H-IoT2023	✓	UDP	DDoS	Binary	CNN	92
[7] (2025)	UL-ECE-MQTT-DDoS-H-IoT2025	✓	MQTT	DDoS	Binary	TCN	99.98
[7] (2025)	UL-ECE-UDP-DDoS-H-IoT2025	✓	UDP	DDoS	Binary	TCN	99.99

While CIC-IDS2017 [12] offers general DDoS coverage, it is primarily designed for intrusion detection based on fundamental network traffic attributes such as timestamps, source and destination IPs, protocols, and attack types. However, it lacks a specific focus on IoT-based scenarios. The dataset is built primarily around protocols like HTTP and HTTPS and includes email-related traffic alongside typical enterprise activities such as web browsing, FTP, and SSH. As a result, it may not adequately capture the complexity of modern IoT and H-IoT systems or reflect the evolving nature of contemporary DDoS attacks targeting such systems.

The BoT-IoT dataset is developed in a simulated IoT environment at the Cyber Range Lab of UNSW Canberra [13]. It contains both benign and botnet traffic, including DoS and DDoS attacks. While it offers a broad range of attack types relevant to IoT, the dataset is fully synthetic and known to be imbalanced [14], with certain classes having very few samples. This imbalance poses challenges for training reliable machine learning models. Moreover, the dataset lacks detailed protocol-level features, which limits its usefulness for analyzing protocol-specific DDoS behaviors in resource-constrained IoT deployments.

The WSN_DDoS_Attack_H-IoT2023 [15] dataset is developed using wireless sensor networks (WSN), focusing on environmental parameters such as humidity and room temperature within healthcare-IoT scenarios. It captures both benign and DDoS traffic over the UDP protocol using the Cooja simulator, representing communication behaviors among sensor nodes under attack conditions. However, a key limitation of this dataset is the absence of direct healthcare sensor data, such as body temperature, heart rate, or other vital signs. Including this simulated data would be representative of actual H-IoT device communications and enhance the dataset’s relevance.

As outlined in Table 5, benchmark datasets like CIC-IDS2017 and BoT-IoT have been widely used to evaluate traditional machine learning models such as Decision Tree (DT) and Random Forest (RF), often yielding high accuracy. The WSN_DDoS_Attack_H-IoT2023 dataset also enables the evaluation of DL models, including Convolutional Neural Networks (CNNs), which are reported to achieve high detection accuracy [3,15]. In this comparison, the TCN model demonstrates improved accuracy over the existing datasets, except for DT, which achieves 100 % on one dataset (e.g., BoT-IoT 2018). These findings underscore the importance of creating protocol-specific datasets tailored for modern IoT and H-IoT DDoS detection research.

## 4 Experimental Design, Materials and Methods

This section presents the data generated through simulation-based experiments using the Cooja and ns-3 simulators. The Cooja simulator generates MQTT-based traffic, while the ns-3 simulator generates UDP-based traffic. Both are configured in a healthcare IoT environment with normal and DDoS-affected nodes. This section also describes simulation design, node configurations, communication protocols, traffic generation behavior, and the tools used to capture and preprocess raw data.

### 4.1 Simulation setup for MQTT

**Fig. 1:**
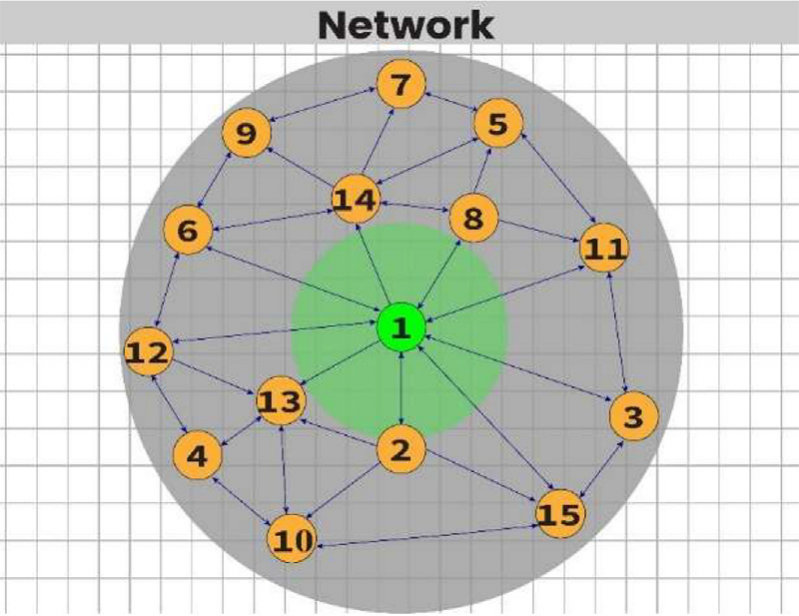
The baseline network topology over the MQTT protocol using the Cooja simulator in an H-IoT environment. The green node represents the MQTT broker/server, and the yellow nodes denote normal H-IoT sensor nodes. This topology diagram is derived from our earlier journal paper [7].

**Fig. 2:**
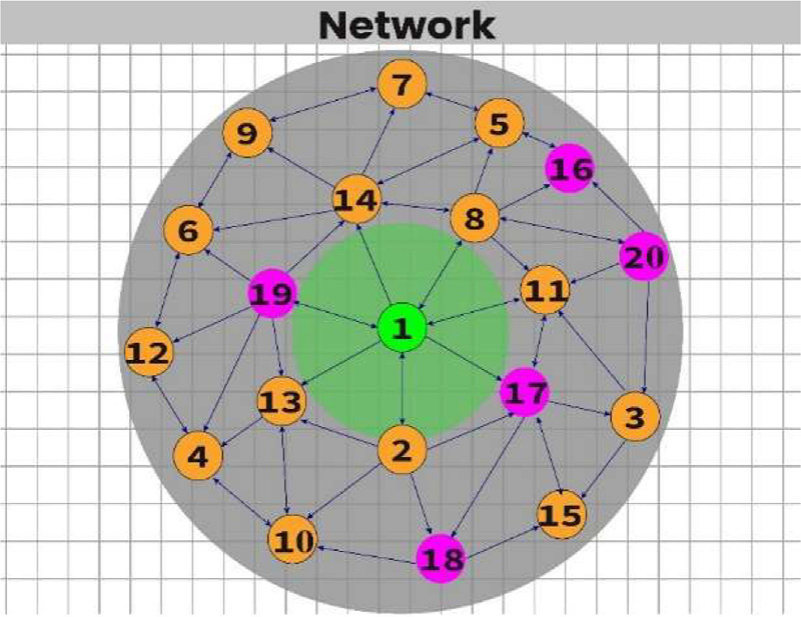
This network topology shows normal and DDoS-affected nodes over the MQTT protocol in the H-IoT environment. The purple nodes indicate DDoS attack-affected nodes. This figure is adapted from our previously published journal paper [7].

The MQTT-based DDoS attack simulation in the H-IoT environment follows the configuration outlined in the related journal paper [7,16]. In brief, the baseline topology consists of 14 emulated H-IoT nodes representing sensors such as body temperature, oxygen saturation, and heart rate, along with one MQTT broker acting as the server, as depicted in Fig. 1. The malicious topology includes five DDoS attack-affected H-IoT nodes, as illustrated in Fig. 2. In the figures, normal nodes are shown in yellow, the MQTT broker (server) is shown in green, and DDoS attack-affected nodes are shown in purple. The simulation is conducted using the Cooja simulator and the Contiki-NG operating system. Normal nodes transmit health-related MQTT messages at regular intervals (e.g., one packet every 60 s), while malicious nodes generate traffic at shorter intervals (e.g., one packet every 20 s) to emulate DDoS behavior. The DDoS-affected nodes transmit data more frequently, every 20 s, to simulate aggressive traffic conditions [7]. Such high-frequency transmissions can degrade server responsiveness. This behavior may also limit the network’s ability to handle legitimate traffic, making it a common technique to disrupt normal operations.

### 4.2 Data generation using Cooja simulator

The synthetic raw data, including regular and malicious traffic, is generated over the MQTT protocol using the Cooja simulator in an H-IoT environment. The data is collected from a simulation of 14 MQTT-enabled sensor nodes emulating three types of healthcare monitoring devices (body temperature, heart rate, and oxygen saturation). Each node transmits data (e.g., a body-temperature reading of 98.6°F) to the server at selected intervals, with different transmission rates assigned to normal and DDoS-affected nodes to reflect distinct communication behaviors. The simulation logs include timestamps, node identifiers, and published MQTT messages, which are captured from the node output console and stored as unprocessed raw text. The simulation runs for 5 h, producing raw data that is saved in a plain text file for further processing.

### 4.3 Simulation setup for UDP

This research simulates a DDoS attack over the UDP protocol in a 5G-enabled H-IoT environment using ns-3, an open-source discrete-event network simulator. A detailed configuration is provided in our related journal publication [7]. The simulation is based on a hierarchical network topology within a 5G-enabled H-IoT environment, which organizes client H-IoT devices, a central server, and multiple LTE base stations (eNodeBs), with communication established via UDP.

**Fig. 3:**
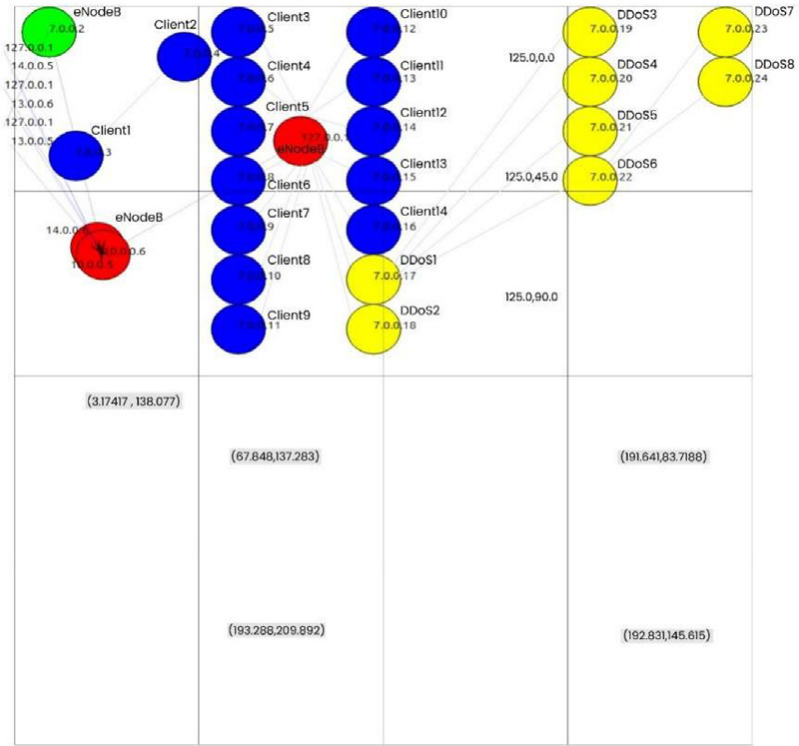
Simulated DDoS attack topology over the UDP protocol using ns-3 in an H-IoT environment. The green node denotes the server, red nodes represent eNodeBs, blue nodes indicate regular H-IoT clients (Client: 1–14), and yellow nodes represent DDoS attackers (DDoS: 1–5). Additional attackers (DDoS: 6–8) are introduced within the same topology to intensify the attack. This figure is derived from the configuration presented in our related journal article [7].

The H-IoT setting includes multiple node types, such as regular client nodes (5–18) generating real-time health-related traffic over the UDP protocol. These nodes are assigned to IP addresses from 7.0.0.3 to 7.0.0.16 and transmit data to the central server (target UE) at IP address 7.0.0.2. DDoS attack-affected nodes (19–23), with IP addresses from 7.0.0.17 to 7.0.0.21, are configured to send high-volume traffic to overwhelm the server, as illustrated in Fig. 3.

Additional DDoS attack-affected H-IoT nodes (24–26, referred to as DDoS-6 to DDoS-8) are dynamically introduced during the simulation to escalate the attack intensity. These nodes, assigned IP addresses from 7.0.0.22 to 7.0.0.24, are activated at 10-second intervals starting 20 s into the simulation. This setup reflects a realistic scenario where new malicious nodes emerge within the same network topology over time. As the number of attacking nodes increases, the central server faces progressively higher volumes of malicious traffic, challenging its capacity to maintain service availability. Evaluating the server's performance under these conditions is essential for assessing its resilience in a 5G-enabled H-IoT environment. Additionally, this scenario facilitates the evaluation of the DL model’s ability to detect newly introduced malicious actors within the same H-IoT topology. The simulation spans 120 s and generates over one million data records, capturing benign and malicious traffic activities.

### 4.4 Data generation using NS-3 simulator

The simulation records detailed communication activity from each H-IoT node to generate raw data, including timestamps, transmitted and received packets, IP and UDP header information, packet sizes, and protocol types. Each entry also captures source and destination IP addresses, port numbers, and payload length. The simulation output includes control-plane messages such as GtpcCreateSessionRequestMessage [17], which represent healthcare device session establishment procedures under a 5G-enabled H-IoT environment. These recorded elements serve as metadata and reflect realistic traffic patterns across various H-IoT nodes, supporting protocol-level inspection and machine learning–based anomaly detection.

## Limitations

The data is not collected from physical H-IoT devices; rather, it is generated through simulations using Cooja and ns-3 simulators. Hence, it may not fully reflect the variability or complexity found in real-world healthcare settings. The attack scenarios and traffic behaviors are predefined based on simulation design, which may not encompass the full range of variability in real-world DDoS events. Moreover, ML/DL models trained on these datasets may require additional hyperparameter tuning (e.g., adjusting learning rate, batch size, or dropout) or further validation with real-world H-IoT data (e.g., testing on actual healthcare network traffic) to enhance their generalizability and robustness in practical deployments.

## Ethics statement

The authors confirm that they have read and complied with the ethical requirements for publication in Data in Brief. This work does not involve human subjects, animal experiments, or data collected from social media platforms.

## CRediT Author Statement

**Mirza Akhi:** Conceptualization; Methodology; Software; Investigation; Data curation; Writing – original draft preparation; Visualization; **Ciarán Eising:** Supervision; Writing – review & editing; **Lubna Luxmi Dhirani:** Supervision; Writing – review & editing.

## Data Availability

ZenodoUL-ECE-MQTT-DDoS-H-IoT2025 and UL-ECE-UDP-DDoS-H-IoT2025 (Original data).
